# Simulation of a field condition to evaluate the risk of enrofloxacin-resistant *Pasteurella multocida* strain selection in food producing rabbits treated via drinking water

**DOI:** 10.3389/fvets.2025.1474409

**Published:** 2025-02-13

**Authors:** Elena Circella, Lorena Lucatello, Ludovica Montanucci, Chiara Belloli, Francesca Capolongo

**Affiliations:** ^1^Department of Veterinary Medicine, University of Bari “Aldo Moro”, Valenzano, Italy; ^2^Department of Comparative Biomedicine and Food Science, University of Padova, Legnaro, Italy; ^3^Department of Neurology, McGovern Medical School, UTHealth, University of Texas Health Science Center at Houston, Houston, TX, United States

**Keywords:** *Pasteurella multocida*, food-producing rabbit, enrofloxacin, medicated water, MSW, PK-PD indices

## Abstract

**Introduction:**

*Pasteurella multocida* is a key bacterial agent involved in most respiratory disorders in rabbits. The objective of this study was to evaluate the risk of selecting *Pasteurella multocida* strains resistant to enrofloxacin (ENRO) in food producing rabbits treated with ENRO via drinking water, according to the standard husbandry practices. Indeed, despite the EU community guidelines recommend a prudent use of antibiotics and promote new strategies to prevent bacterial diseases, antimicrobial therapy remains the primary approach for pasteurellosis management in rabbits. Therefore, the potential risk of selecting resistant bacteria in food-producing animals requires identifying optimized dosage regimens to minimize resistance emergence and to extend the useful lifetime of the drug.

**Methods:**

In this study, we isolated *Pasteurella multocida* strains from bacterial colonies sampled in nasal swabs collected from 6 healthy rabbits and 12 rabbits suffering respiratory disorders. Animals were sourced from industrial farms and were randomly selected to investigate the inter-individual variability in antimicrobial exposure associated with treatment via drinking water. Sick rabbits underwent an approved ENRO treatment (10 mg/kg for 5 days) administered via drinking water, following standard husbandry practices. We investigated the minimum inhibitory concentration (MIC), the minimum bactericidal concentration (MBC), and the mutant prevention concentration (MPC) of ENRO against bacterial strains in healthy rabbits and in sick rabbits before and after treatment. We recorded plasma drug concentrations of treated animals, and we applied the mutant selection window (MSW) approach to each subject. Finally, we calculated the PK/PD indices for concentration-dependent antimicrobials to assess ENRO’s clinical efficacy and it’s potential for promoting resistance using published pharmacokinetic (PK) parameters and maximum drug plasma concentrations recorded in this study.

**Results:**

Here we showed that treatment with ENRO improved clinical signs in rabbits with pasteurellosis but failed to completely eradicate the pathogen, consistent with previous studies. MPC-based analysis showed acquired resistance and potential ENRO-induced shift to a lesser sensitivity in the *P. multocida* population. Moreover, MSW analysis revealed that 45% of treated rabbits exhibited potential for drug resistance selection.

**Conclusion:**

These findings suggest that the current ENRO dosing regimen for pasteurellosis in rabbits is inadequate and may contribute to resistance development.

## 1 Introduction

Infectious upper respiratory disease commonly known as “snuffles” is considered the most prevalent disease in food-producing, pet, and laboratory rabbits (1, 2). *Pasteurella multocida* (*P. multocida*) is one of the main bacterial agents associated with respiratory disorders and the primary or secondary pathogen in the commensal respiratory tract microbiota. Therefore, the respiratory form of pasteurellosis in rabbits refers to a respiratory syndrome in which *P. multocida* coexist with other bacteria (e.g., *Bordetella bronchiseptica, Staphylococcus areus*) (2–4). In food-production rabbits, pasteurellosis is a major cause of economic losses worldwide, and its prevention and control primarily rely on effective biosecurity measures (such as avoiding overcrowding and adopting good facilities and adequate hygiene practices) and on vaccination (2, 5). However, curative and metaphylactic antimicrobial therapies remain the most commonly employed strategies for infection management.

The main classes of antibiotics approved for treating pasteurellosis include *Critically Important Antimicrobials* (CIAs), such as fluoroquinolones (6, 7). These drugs, classified as highest-priority for managing antimicrobial resistance risks, have demonstrated efficacy in treating various animal diseases, including rabbit “snuffles” (8–10). Unfortunately, due to their widespread use and misuse, many *P. multocida* isolates have developed resistance to fluoroquinolone, raising concerns about the emergence of resistant bacteria in food-producing animals (11). This highlights the importance of methods aimed at minimizing resistance emergence and preserving the efficacy of this antimicrobial class.

One such method is antibiotic dose optimization. *In vitro* studies simulating current dosing practices have shown that drug exposures sufficient to increase the probability of clinical cure may still be insufficient to suppress the emergence of antibiotic-resistant Gram-negative bacteria (12). Reducing the likelihood of mutant selection during therapy could be achieved using dosing regimens capable of inhibiting not only the susceptible bacteria but also the sub-population of resistant mutants.

Enrofloxacin (ENRO), a second-generation fluoroquinolone exclusively used in veterinary medicine (10), is routinely administered to rabbits via drinking water. Currently, *P. multocida* exhibits low resistance to ENRO in this animal species, with a reported resistance rate of 0.2% (11, 13, 14). Optimizing ENRO dosing regimens is therefore of paramount importance for extending the effective lifespan of this antimicrobial drug in rabbits.

At present, there are no standardized practices for determining antibiotic exposures that are optimal for suppressing or minimizing the emergence of antibiotic resistance against different bacteria strains. However, published research has increasingly recognized the importance of the pharmacokinetic/pharmacodynamics (PK/PD) approach in designing dosing regimens that are effective both clinically and in minimizing resistance emergence. This approach can potentially be applied across individuals and animal species (15).

The PK/PD indices relate the antibiotic exposure of a pathogen to its sensitivity to the drug, providing clinicians with a dosing target (15, 16). For concentration-dependent antimicrobials like ENRO, key indices include AUC_24_/MIC, which is the ratio of the area under the drug concentration-time curve (AUC_24_, which reflects total antibiotic exposure), to the minimum inhibitory concentration (MIC), and C_max_/MIC, which is the ratio of the maximum drug concentration (C_max_) to the minimum inhibitory concentration (MIC) (17, 18). These indices, which are based on the MIC as a measure of bacterial susceptibility, provide a robust foundation for defining clinically effective dosages. Alternative parameters focused on defining the potential for resistance development, such as the mutant prevention concentration (MPC), have also been proposed (19, 20). MPC, conceptualized in 1999 (21), is the antibiotic concentration required to suppress the growth of first-generation mutant bacteria that may selectively proliferate at concentrations above the MIC. This concept aligns with the mutant selection window (MSW) hypothesis, which states that concentrations within the range between the MIC and the MPC (i.e., the MSW), selectively enrich resistant bacteria (22).

However, the universal applicability of the PK/PD approach is limited by the large inter-individual heterogeneity in antimicrobial exposure associated with oral treatments via drinking water (23). Even if only a small percentage of treated individuals are exposed to drug concentrations within the MSW (hence suitable for the selection of resistance genes) this could pose a risk of spreading drug resistance. Hence, evaluating individual drug exposure within treated groups is essential.

This study aimed to assess changes in PD parameters values following ENRO treatment under field conditions and to determine whether blood drug concentrations achieved during treatment align with clinical efficacy requirements and with those needed to minimize resistance development to ENRO.

To this end, the PD parameters (MIC, MBC, MPC) of ENRO against *P. multocida* strains were evaluated in samples collected from healthy rabbits and from rabbits with respiratory disorders before and after an approved ENRO treatment. The treatment was administered via drinking water according to standard husbandry practices and following the instructions reported in the leaflet of the approved pharmaceutical medication (10 mg/kg for 5 days). During treatment, plasma drug concentrations were recorded to verify their consistency with the sensitivity profiles of the *P. multocida* strains isolated from individual subjects. Finally, we assessed whether the authorized dosage of ENRO for oral administration (10 mg/kg) poses a risk of selecting drug-resistant strains in rabbits when delivered via drinking water. This administration route may fail to achieve plasma concentrations necessary to control resistance emergence. To address this, we calculated PK/PD indices for concentration-dependent antimicrobials integrating the recorded PD parameters, PK parameters derived from literature (24), and the maximum drug plasma concentrations recorded in this study.

## 2 Materials and methods

### 2.1 Chemicals and reagents

Quinoflox (100 mg/mL, Global vet Health, S.L. – Spain). Enrofloxacin (ENRO, purity: 99.0%), ciprofloxacin (CIPRO, purity: 99.9%) and norfloxacin (internal standard - IS, NOR, purity: 99.7%) were obtained from Sigma Aldrich (Steinheim, Germany). Enrofloxacin (Bayer Animal Health, Milano, Italy). Acetonitrile (ACN) and methanol (MeOH) were purchased from Carlo Erba Reagents (Carlo Erba, Milan, Italy). Formic acid (FA, 98%), ammonium acetate (98%) and potassium phosphate monobasic KH_2_PO_4_ were obtained from Sigma Aldrich (Steinheim, Germany). All reagents were of analytical grade. Ultrapure water was generated using a Milli-Q system (Millipore). Phenex-RC (regenerated cellulose) syringe filters 0.22 mm (Phenomenex, Torrance, CA, United States) were used to filter the extracts before injection into the LC–MS system.

### 2.2 Animals

The study was conducted on 6 healthy female rabbits (Group A: A1-A6) with a mean body weight of 3.6 kg (±0.1 s.e.m.) and 12 female rabbits (Group B: B1-B12) weighting 4.1 kg (±0.2 s.e.m.) affected by respiratory disorders (“snuffling rabbits”) of varying severity (rhinitis, sneezing, muco-catarrhal nasal discharge, epiphora). The health status of the rabbits was determined through a thorough clinical examination. Group A rabbits were sourced from an industrial farm where the disease had been controlled for approximately 4 years using an inactivated autovaccine against *P. multocida*. Group B rabbits were selected from two industrial farms (B1- B6 and B7-12, respectively) where no vaccine against *P. multocida* was used and cases of “snuffles” were frequently reported. To replicate field conditions and to investigate inter-individual variability in antimicrobial exposure, animals were randomly selected from farms located in southern Italy (Apulia and Basilicata). This study complied with current animal welfare regulations (Directive 98/58/EC and Italian Decree Law 146/2001) and was approved by the Institutional ethical committee for Animal Research of Veterinary Medicine Department of University of Bari (Italy) (approval n°2/16).

### 2.3 Experimental design

#### 2.3.1 Trials in healthy rabbits (group A)

Following a 3-day acclimation period, the water intake of healthy rabbits was measured. Each rabbit had unrestricted access to tap water provided in a plastic sipper bottle throughout the study. The water volume (mL), was replenished daily at 08:00 a.m. and monitored at 02:00 p.m., 07:00 p.m. and again the following morning at 08:00 a.m. before replacement. Drinking water intake (mL/day or mL/kg/day) was calculated by determining the difference between the recorded volume of water at each time point and the volume measured at previous time point. On the day prior to initiating water intake measurements, a nasal swab was collected from each animal’s nasal cavity to assess the commensal population of *P. multocida*.

#### 2.3.2 Trials in sick rabbits (group B)

Following the clinical examination and diagnosis of “snuffles,” ENRO (Quinoflox 100 mg/mL, Global vet Health, S.L. - Spain) was administered via medicated water to the 12 sick rabbits. The commercial product was diluted in fresh water according to the manufacturer’s instructions to deliver a dose of 10 mg ENRO/kg body weight (mL medicine/L H_2_O = [0.1 mL medicine (100 mg/mL) × mean animal BW (kg) × number of animals] / total water intake during 24 h before treatment initiation). The medicated water was prepared daily, based on the mean weight of 4.1 kg (± 0.2 s.e.m.) and on the total water consumed daily by all rabbits (3.44 L), as recorded the day prior to treatment initiation. The actual individual drug dosage (mg/kg) for each rabbit was calculated by considering two factors: the total daily water consumption per kilogram of body weight (mL/kg), and the ENRO concentration in the drinking water.

The medicated water, freshly diluted, was provided *ad libitum* to each rabbit at 08:30 a.m. for 5 days in a plastic sipper bottle. Throughout the treatment period, starting from the day before its initiation, the rabbits´ daily water intake was monitored before fresh medicated water was offered. Water collection and measurements were performed as in Group A. Starting on day 2 of treatment, blood samples were collected by venipuncture in EDTA tubes from the lateral saphenous vein of each rabbit. Sampling times were chosen based on the drinking behavior observed in Group A rabbits, to capture the peak and nadir drug concentrations in the bloodstream. In order to minimize stress and avoid disrupting normal drinking patterns, blood sampling was limited to two per day for each rabbit. Sampling was performed daily at 08:30 a.m., immediately before the fresh medicated water was offered and at 02:00 p.m., corresponding to the times of maximum and minimum water intake, respectively. Plasma was separated by centrifugation and stored at −20°C until analysis for ENRO and its active metabolite, ciprofloxacin (CIPRO).

To detect *P. multocida* in the nasal cavity, nasal swabs were collected from each rabbit the day before treatment initiation and 3 days after the end of the treatment.

### 2.4 Bacterial strain isolation

*Pasteurella multocida* isolation was carried out using standard cultural methods. Briefly, nasal swabs were plated on blood agar (TSA - Tryptic Soy Agar, Oxoid Ltd., Basingstoke, United Kingdom), supplemented with 5% sheep blood and incubated at 37°C for 24 h. Blood agar allows the growth of various bacteria, some of which may or may not be associated with respiratory disorders, however in this work we only isolated *P. multocida*. To obtain pure cultures, *P. multocida*-suspected colonies were therefore plated again under the same culture conditions.

Three to five colonies from each plate were tested using PCR, following the protocol described by Townsend et al. (25), to confirm the presence of *P. multocida*. PCR amplification products were analyzed via gel electrophoresis on 1.5% agarose gel, stained with ethidium bromide and visualized using the Gel Doc-It image analyzer (UVP, Upland, CA, United States). All isolates from each nasal swab were pooled and stored at −80°C in Brain Heart Infusion broth (BHIB - Oxoid Ltd., Basingstoke, United Kingdom) until antimicrobial susceptibility tests were conducted.

### 2.5 *In vitro* antimicrobial susceptibility testing

The ENRO (Bayer Animal Health, Milano, Italy) stock solution was prepared in methanol - NaOH 1 N mixture (20:80 - v: v; pH 13.4) at a concentration of 10 mg/mL and stored at room temperature (20–22°C), protected from light. Water dilutions suitable for the assays were freshly prepared on each experimental day. The drug concentration was verified spectrophotometrically (spectrophotometer Beckman DU640 - Beckman Coulter, Brea, United States) at a wavelength of *λ* = 272 nm (26).

#### 2.5.1 Minimum inhibitory concentration

The MIC assays were performed using the broth microdilution method as described in the CLSI guidelines, with minor modifications (27). The assays were performed in Mueller–Hinton broth (Oxoid Ltd., Basingstoke, United Kingdom) adjusted with Ca^++^/Mg^++^ and supplemented with 5% lysed sheep blood (CAMHB-LSB). The inoculum concentration (5 × 10^5^ CFU/mL in well) was verified spectrophotometrically at λ625 using a Beckman DU64a spectrophotometer and was confirmed by plate counting after serial dilutions. *Escherichia coli* ATCC 25922 served as quality control strain. Serial two-fold dilutions of antimicrobial agents were tested at concentrations ranging from 0.002 and 256 μg/mL. Each well of the 96-well plates was filled with drug, media and culture and then statically incubated under aerobic conditions at 35°C overnight (approximately 20 h). Each isolate was tested at least three times in triplicate, alongside a positive control (medium and pathogen only), a negative control (medium and drug solvent) and a blank (medium only). The MIC was determined as the lowest drug concentration that inhibited visible bacterial growth after incubation.

The reproducibility of MIC assays was deemed acceptable within one two-fold dilution. Actual intra- and inter-test MIC endpoints were rounded up to the highest concentration recorded.

In the absence of ENRO clinical susceptibility breakpoints for *P. multocida* in rabbits, the bacterial sensitivity to ENRO of the isolated strains was classified using CLSI clinical breakpoints established for swine respiratory disease (sensitive: MIC ≤0.25 μg/mL; intermediate: MIC = 0.5 μg/mL; resistant: MIC ≥2 μg/mL) and for bovine, poultry and turkey respiratory diseases (sensitive: MIC ≤0.25 μg/mL; intermediate: MIC 0.5–1 μg/mL; resistant: MIC ≥2 μg/mL) (28, 29).

#### 2.5.2 Minimum bactericidal concentration

The MBC assays were performed following the NCCLS guidelines (30). The well contents corresponding to the MIC and to the three higher subsequent concentrations were collected from the plates of the MIC assays previously carried out and plated in duplicate on CAMH agar (Oxoid Ltd., Basingstoke, United Kingdom) supplemented with 5% sheep blood (CAMHA-SB).

After overnight incubation at 35°C, the MBC was defined as the lowest concentration that reduced bacterial numbers by 99.9%. Each data point was derived from at least three independent experiments. The MBC rejection value was determined according the NCCLS guidelines (30, 31), and the bactericidal index (BI = MBC/MIC) was calculated for each isolate and drug combination.

#### 2.5.3 Mutant prevention concentration

The MPC experiments were performed as previously described by Marcusson et al. (32), with minor modifications.

To obtain a high-density pure suspension of *P. multocida*, 30 μL of the frozen stock suspension was inoculated on blood agar plates (TSA, Biolife Italiana Srl, Milan, Italy, supplemented with 5% sheep blood) and incubated overnight at 37°C.

A portion of the colonies was then incubated in BHIB (Oxoid Ltd., Basingstoke, United Kingdom) supplemented with 5% lysed sheep blood (BHIB-LSB) at 35°C for 3 h. The colony suspension was then diluted to 1:100 and plated onto blood agar plates to obtain well-isolated single colonies with the typical *P. multocida* morphology after incubation for 24 h at 35°C.

Four colonies were seeded onto two blood agar plates each and further incubated for 24 h at 35°C. All bacterial growth obtained was suspended in 5 mL of BHIB-LSB and incubated again for 24 h at 35°C. Finally, 1 mL of culture was transferred into 50 mL of BHIB-LSB and incubated at 35°C for 4 h to obtain an OD_540_ of ⁓ 1, corresponding to ⁓ 10^9^ CFU/mL. Bacterial density was confirmed by plate counting. Aliquots of 10 mL of cultures were centrifuged at 1,500 x g for 15 min, and the supernatant was discarded. The pellets (approximately 10^10^ CFU) were re-suspended in the remaining broth, spread on CAMHA-SB plates containing serial concentrations of the drug tested (1, 2, 4, 8, 16 and 32xMIC) and incubated at 35°C for 72 h. The MPC was defined as the lowest drug concentration at which no colonies were found; it was determined for each strain at least in three independent experiments.

The MSW (MPC-MIC) and the mutant prevention index (MPI = MPC/MIC) were calculated for each strain.

### 2.6 LC–MS/MS plasma drug concentration analysis

Liquid chromatography coupled to mass spectrometry (LC–MS/MS) was applied to identify and quantify ENRO and CIPRO in plasma from rabbits, as described in Lucatello et al. (33). Chromatographic separation was performed using an Accela 600 HPLC pump with a CTC automatic injector (Thermo Fischer Scientific, San Jose, CA, United States), equipped with a C-18 Kinetex (100 × 2.1 mm, 2.6 μm) analytical column by Phenomenex (Torrence, United States). Mass spectrometric analysis was performed using an LTQ XL ion trap (Thermo Fischer Scientific, San Jose, CA, United States) equipped with a heated electrospray ionization (HESI-II) source controlled by the Xcalibur software (version 2.1, Thermo Electron Corporation). Plasma sample purification was performed by protein precipitation with acetonitrile (33). Briefly, a volume of 3 mL of ACN was added to 200 μL of rabbit plasma fortified with 10 μL of internal standard (IS) solution (0.5 mg/L). The tubes were shaken for 10 min using a horizontal mechanical agitator and then centrifuged at 4,000 rpm for 10 min. The supernatant was transferred to a 5-mL tube and evaporated to dryness under an air stream at 50°C using a Turbovap evaporator (Zymark, Hopkinton, MA, United States). The residue was reconstituted with 200 μL of the mobile phase (0.1% formic acid in ammonium acetate 10 mM, pH 2.5: 0.1% formic acid in MeOH; 80%:20%, v: v). Finally, Phenex-RC syringe filters (0.22 μm) were used to filter the extracts prior to injection into the LC–MS/MS system. The plasma ENRO and CIPRO LOQ values were 2.5 and 1.4 μg/L, respectively.

### 2.7 Data analysis

The Wilcoxon matched pairs test was applied to compare MICs versus MPCs before and after treatment, respectively. The Mann–Whitney test was used to assess potential differences in ENRO potency of strains collected from different farms, between healthy and sick animals, before and after treatment. The Student’s *t*-test was applied to evaluate differences in water intake, between healthy and sick animals, as well as between night and day. Difference was considered statistically significant when the *p*-value was less than 0.05 (*p* < 0.05).

The PK/PD indices for clinical efficacy (AUC_24_/MIC and C_max_/MIC) and for controlling drug resistance (AUC_24_/MPC and C_max_/MPC) were calculated by integrating the PD parameters recorded in this study with the PK parameters derived from literature (24) or from the maximum mean daily concentration observed during a four-day treatment period.

## 3 Results

The ENRO, administered via drinking water (10 mg/kg) for 5 days, improved symptoms in rabbits affected by mild respiratory disease but did not result in significant symptomatic changes in rabbits affected by more severe respiratory disorders.

### 3.1 *In vitro* antimicrobial susceptibility testing

Table 1 shows the *in vitro* antibacterial activity of ENRO against six *P. multocida* clinical isolates from healthy rabbits and 12 isolates from rabbits affected by respiratory disorders before treatment.

**Table 1:** Minimum inhibitory concentration (MIC) and minimum bactericidal concentration (MBC) values for 18 individual *Pasteurella multocida* strains isolated from 6 healthy rabbits (A) and 12 rabbits suffering respiratory disorders from two different farms (B).

Isolates	MIC* μg/mL	MBC μg/mL	BI
A1	0.03	n.d	–
A2	0.015	0.03	2
A3	0.125	0.25	2
A4	0.03	n.d	–
A5	0.125	0.25	2
A6	0.03	n.d	–
B1	0.125	0.25	2
B2	0.125	0.25	2
B3	0.03	0.03	1
B4	0.06	0.125	2
B5	0.015	n.d	–
B6	0.125	0.25	2
B7	0.03	0.06	2
B8	0.004	0.004	1
B9	0.004	0.008	2
B10	0.03	0.06	2
B11	0.03	0.06	2
B12	0.03	0.06	2

n.d. =not determined. *Data were obtained from three experiments performed at least in triplicate and the acceptable reproducibility of values was set within one two-fold dilution. No significant differences were observed in susceptibility to enrofloxacin between healthy and sick animals and among farms of origin (Mann–Whitney test). BI (bactericidal index) = MBC/MIC ratio.

**Figure 1:**
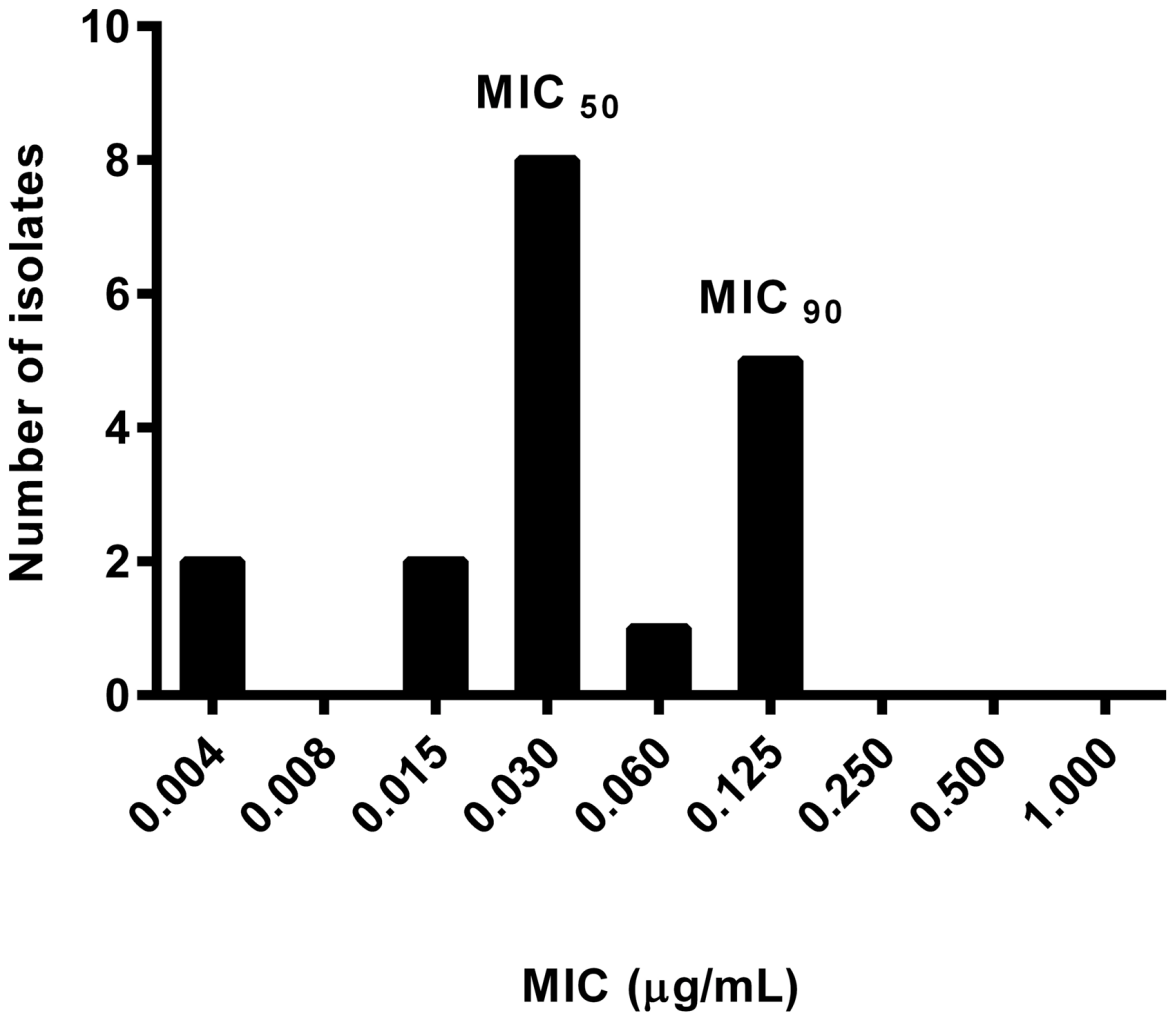
Enrofloxacin minimum inhibitory concentration (MIC) distribution of the 18 *Pasteurella multocida* strains isolated from 6 healthy rabbit and 12 rabbits suffering respiratory disorders.

No significant differences in susceptibility to ENRO were observed either between healthy and sick animals or among farms. Therefore, the following analyses were carried out considering all tested strains, globally. The ENRO MIC distribution for 18 *P. multocida* strains is shown in Figure 1. The ENRO MIC values ranged from 0.004 to 0.125 μg/mL, and the shape of the distribution suggests a bimodal trend. The drug concentrations inhibiting 50 and 90% of bacterial strains, i.e., MIC_50_ and MIC_90_, were estimated at 0.03 and 0.125 μg/mL, respectively.

The bactericidal concentration of ENRO was reached at 2 x MIC in 86% of strains and at MIC concentration in 14% of the tested strains (Table 1).

The MICs and MPCs of ENRO for the *P. multocida* strains isolated from sick rabbits before and after treatment with ENRO (10 mg/kg) via drinking water are presented in Table 2.

**Table 2:** Minimum inhibitory concentration (MIC) and mutant prevention concentration (MPC) of enrofloxacin against *Pasteurella multocida* strains isolated from rabbits suffering respiratory disorders before and after treatment with enrofloxacin (10 mg/kg) *via* medicated water.

Strains	MIC^a*^ μg/mL	MPC^b^ μg/mL	MSW μg/mL	MPI	MIC^a*^ μg/mL	MPC^b^ μg/mL	MSW μg/mL	MPI
	before treatment				after treatment			
B1	0.125	n.d.	–	–	n.d.	n.d.	–	–
B2	0.125	0.5	0.374	4	0.125	1	0.875	8
B3	0.03	0.125	0.095	4.2	0.015	n.d.	–	–
B4	0.06	n.d.	–	–	n.d.	n.d.	–	–
B5	0.015	0.125	0.11	8.3	0.015	0.25	0.235	16.7
B6	0.125	0.5	0.375	4.0	0.25	1	0.75	4.0
B7	0.03	0.5	0.47	16.7	0.015	0.25	0.235	16.7
B8	0.004	0.03	0.026	7.5	0.004	0.03	0.026	7.5
B9	0.004	0.06	0.056	15.0	0.004	0.25	0.246	62.5
B10	0.03	0.25	0.22	8.3	0.25	1	0.75	4.0
B11	0.03	0.25	0.22	8.3	0.004	0.125	0.121	31.3
B12	0.03	0.25	0.22	8.3	0.06	0,5	0.44	8.3

n.d. = not determined. ^a,b^ = P < 0.001, Wilcoxon matched pairs test. *Data were obtained from three experiments performed at least in triplicate and the acceptable reproducibility of values was set within one two-fold dilution. MSW (mutant selection window) = MPC-MIC; MPI (mutant prevention index) = MPC/MIC ratio.

For strains collected before treatment, the MIC and MPC values ranged from 0.004 to 0.125 μg/mL and from 0.03 to 0.5 μg/mL, respectively. For strains collected after treatment, these values ranged from 0.004 to 0.25 μg/ mL and from 0.03 to 1.0 μg/mL, respectively. No statistical differences were observed when comparing MICs before and after treatment, whereas significant differences were observed when comparing the MIC and MPC values recorded before and after treatment (*p* < 0.001). The MPI values for the pre-treatment strains ranged from 4 to 16.7, and those for the post-treatment strains ranged from 4 to 62.5.

**Figure 2:**
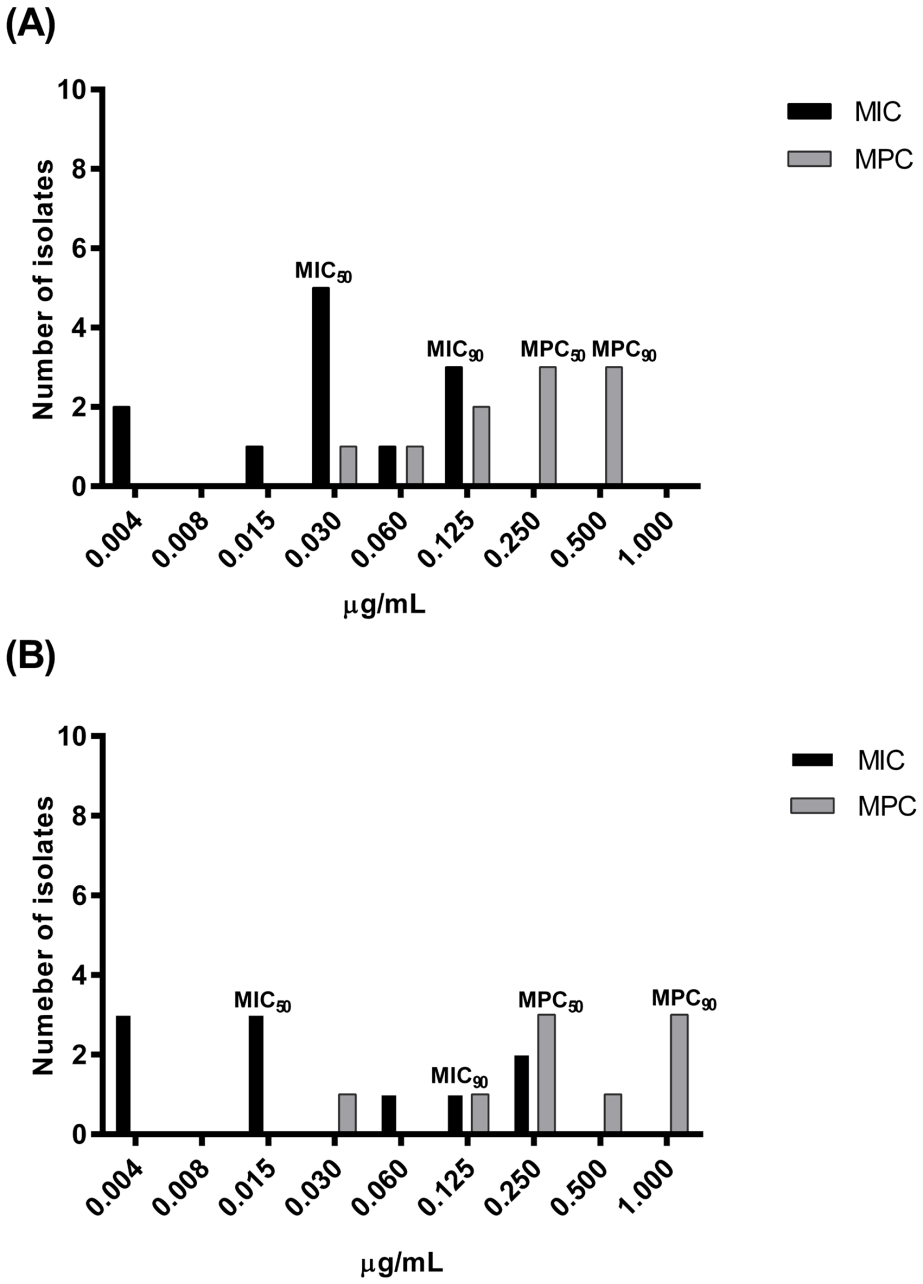
Enrofloxacin minimum inhibitory concentration (MIC) and mutant prevention concentration (MPC) distribution of pathogenic *Pasteurella multocida* strains isolated from 12 rabbits suffering respiratory disorders before **(A)**: MIC, *n* = 12; MPC, *n* = 10 and after treatment **(B)**: MIC, *n* = 10; MPC, *n* = 9 with enrofloxacin (10 mg/kg) administered via medicated water.

The MIC and MPC distributions, as well as the MIC_50_ and MIC_90_ or MPC_50_ and MPC_90_, detected before and after-treatment are presented in Figures 2A,B, respectively. The MPC_50_ increased approximately 8 times the corresponding MIC_50_ before treatment and 16 times after treatment, while the MPC_90_ increased from 4 to 8 times for strains collected before or after treatment, respectively.

### 3.2 Drug plasma concentration analysis

#### 3.2.1 Medicated water intake

The mean daily water consumption is reported in Table 3. Rabbit water intake showed no significant variation between day and night. Additionally, no significant differences in water intake were observed between healthy and sick animals receiving ENRO-treated drinking water.

**Table 3:** Mean water daily (healthy or sick treated animals with enrofloxacin 10 mg/kg, administered via drinking water for 5 days) and day/night consumption (all animals) over 5 days recorded.

	Weight	Mean daily water consumption	
	(kg ± s.e.m)	(mL/day ± s.e.m)	(mL kg/day ± s.e.m)
Healthy animals (*n* = 6)	3.5 ± 0.1	283 ± 18.0	81 ± 18.5
Sick animals (*n* = 12)	4.1 ± 0.2	239 ± 6.0	59 ± 5.4

	Mean day/night water consumption	
	(mL/h, day* ± s.e.m)	(mL/h, night** ± s.e.m)
All animals (*n* = 18)	12.0 ± 5.0	10.2 ± 1.5

*day: from 08:00 a.m to 07:00 p.m.; ** night: from 07:00 p.m to 08.00 a.m.

Based on the daily water intake and following the dilution instructions provided in the medicine leaflet, the rabbits received an average dose of 10.4 ± 0.8 mg/kg, with individual doses ranging from a minimum of 7.6 mg/kg to a maximum of 15.4 mg/kg (Table 4).

**Table 4:** Actual enrofloxacin (ENRO) individual dosage (mg/kg) and mean (± s.e.m.) plasma concentrations of ENRO, its metabolite ciprofloxacin (CIPRO) and their sum (ENRO+CIPRO), recorded in samples collected twice a day (08:30 a.m. and 02:00 p.m.) in 12 rabbits suffering respiratory disorders and treated with ENRO administered *via* medicated water according to the manufacturing instructions.

		08:30 a.m.			02:00 p.m		
Animal	Dosage mg/kg	ENRO μg/mL	CIPRO μg/mL	ENRO+CIPRO μg/mL	ENRO μg/mL	CIPRO μg/mL	ENRO+CIPRO μg/mL
B1	7.76	0.034 ± 0.004	0.021 ± 0.001	0.056 ± 0.008	0.036 ± 0.004	0.020 ± 0.002	0.056 ± 0.005
B2	11.89	0.076 ± 0.006	0.038 ± 0.002	0.114 ± 0.015	0.065 ± 0.006	0.031 ± 0.003	0.096 ± 0.008
B3	9.81	0.023 ± 0.001	0.018 ± 0.001	0.041 ± 0.004	0.033 ± 0.005	0.025 ± 0.003	0.059 ± 0.008
B4	5.68	0.060 ± 0.011	0.035 ± 0.010	0.099 ± 0.041	0.063 ± 0.011	0.032 ± 0.006	0.094 ± 0.015
B5	8.85	0.040 ± 0.009	0.023 ± 0.001	0.063 ± 0.019	0.036 ± 0.005	0.020 ± 0.001	0.056 ± 0.006
B6	15.57	0.220 ± 0.035	0.053 ± 0.010	0.276 ± 0.084	0.243 ± 0.028	0.059 ± 0.007	0.301 ± 0.034
B7	10.51	0.172 ± 0.029	0.070 ± 0.017	0.242 ± 0.092	0.144 ± 0.022	0.059 ± 0.010	0.203 ± 0.030
B8	13.47	0.114 ± 0.027	0.063 ± 0.015	0.176 ± 0.084	0.114 ± 0.020	0.065 ± 0.010	0.179 ± 0.030
B9	9.27	0.108 ± 0.011	0.041 ± 0.002	0.150 ± 0.026	0.100 ± 0.012	0.039 ± 0.003	0.140 ± 0.015
B10	8.79	0.220 ± 0.020	0.040 ± 0.004	0.260 ± 0.049	0.177 ± 0.021	0.036 ± 0.003	0.213 ± 0.023
B11	8.75	0.152 ± 0.024	0.045 ± 0.002	0.196 ± 0.052	0.189 ± 0.027	0.053 ± 0.005	0.242 ± 0.032
B12	10.23	0.069 ± 0.009	0.027 ± 0.002	0.097 ± 0.021	0.074 ± 0.007	0.028 ± 0.002	0.102 ± 0.009
Mean	10.01	0.108	0.040	0.148	0.106	0.039	0.145
s.e.m	0.75	0.020	0.005	0.024	0.020	0.005	0.024

#### 3.2.2 LC MS/MS analysis

Both the parent drug (ENRO) and its metabolite (CIPRO, formed through the metabolic conversion of ENRO) were detected. High variability in plasma drug concentration was observed, ranging from 0.041 to 0.276 μg/mL for the sum of ENRO+CIPRO. Contrary to expectations, no correlation was found between the dose taken by individual subjects and the plasma concentration of the drug.

**Figure 3:**
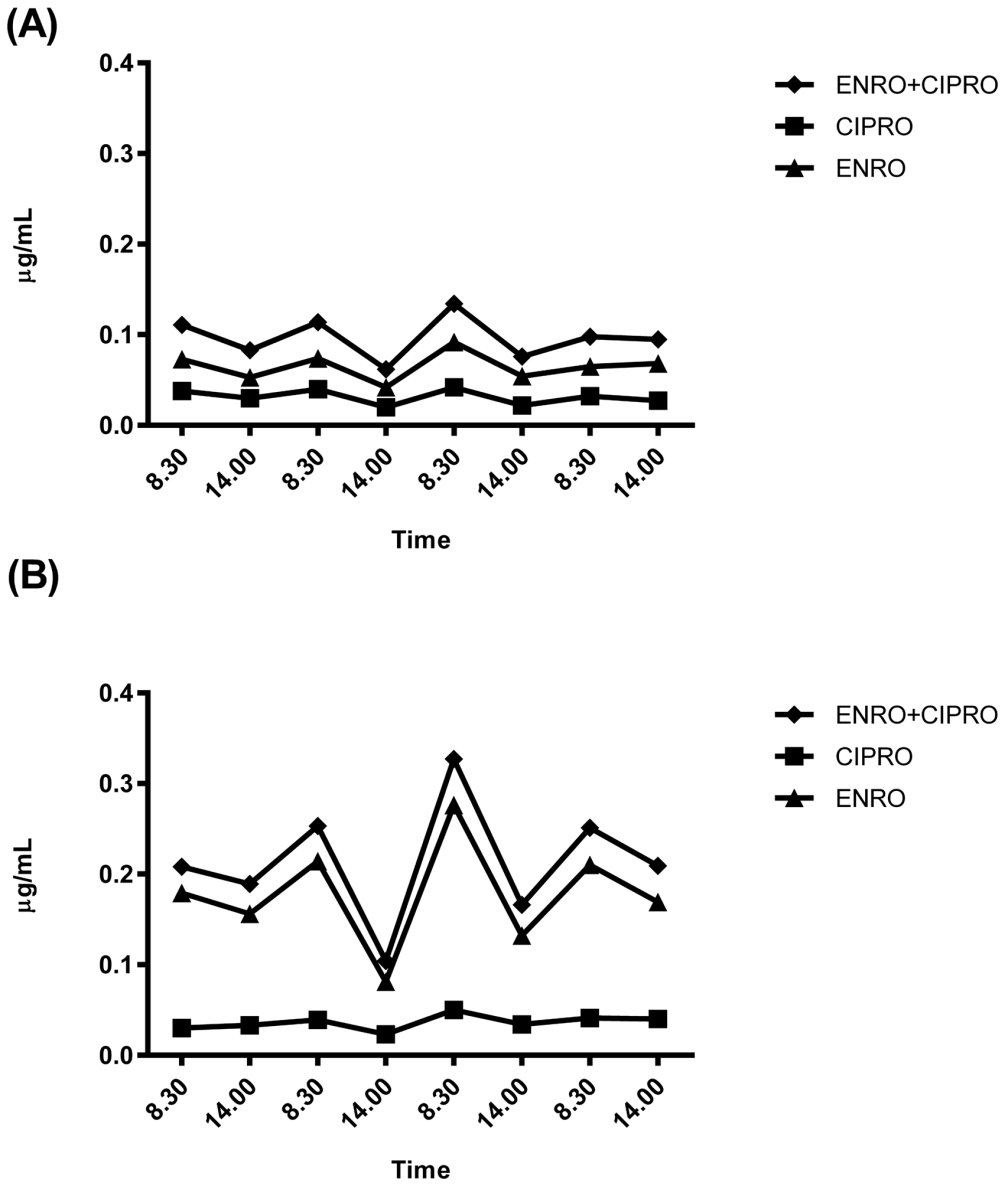
Representative plasma concentration-time profiles of enrofloxacin (ENRO) and its metabolite ciprofloxacin (CIPRO) recorded in the two rabbits showing the lower **(A)** and higher **(B)** plasma drug concentrations recorded.

Figures 3A,B show the plasma concentration-time profiles of ENRO and its metabolite CIPRO in the two rabbits with the highest and lowest plasma drug concentrations.

The plasma concentration profiles consistently revealed higher concentrations in the morning (08:30 a.m. - peak) than at 02:00 p.m. (nadir). However, no significant differences between morning and evening sampling time points were detected, with the mean fluctuation width being approximately 0.03 μg/mL as ENRO+CIPRO.

The peak plasma concentration of ENRO, of its metabolite CIPRO and of their sum (ENRO+CIPRO) recorded for the 12 treated rabbits are presented in Table 4.

**Figure 4:**
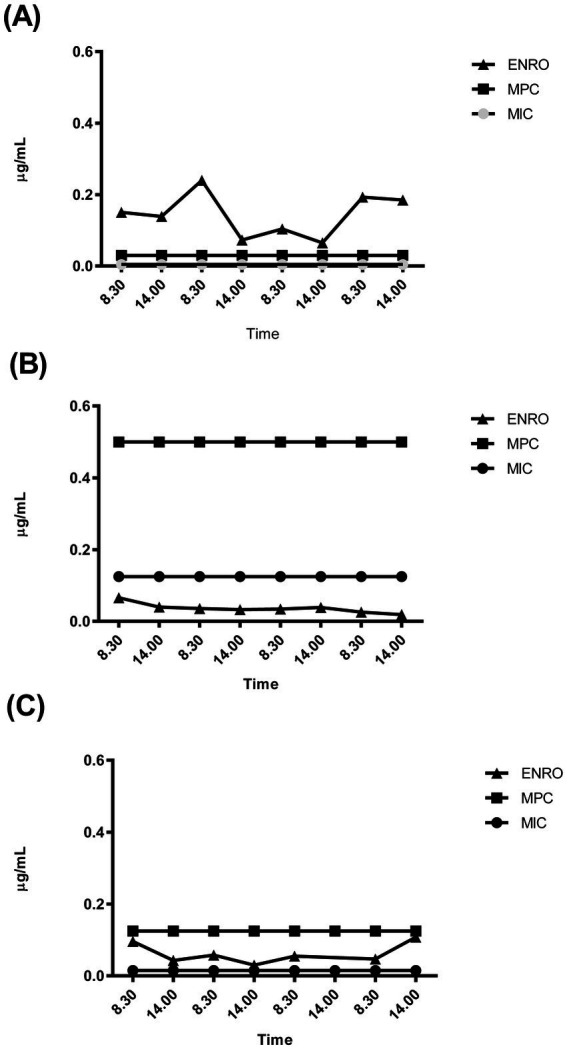
Plasma concentration-time profile of enrofloxacin (ENRO) recorded for the three representative rabbits whose trends were above **(A)**, below **(B)** or within **(C)** the mutant selection window. MIC (minimum inhibitory concentration = lower limit); MPC (mutant prevention concentration = upper limit).

Individual analysis of the ENRO concentration trend compared to that of the corresponding MSW was conducted for nine rabbits. Treatment with ENRO resulted in plasma levels higher than the MPC in two subjects (B8, B9), lower than the MIC or borderline in three subjects (B2, B3, B6) and within the MSW in four subjects (B5, B10, B11, B12) (Figures 4A–C).

Table 5 presents the PK/PD indices of ENRO, calculated using the recorded MIC_50_ and MPC_50_ values, and the PK parameters derived from the literature. Additionally, the table includes the PK/PD index of ENRO calculated based on the highest mean plasma concentrations of ENRO+CIPRO recorded in this study.

**Table 5:** Pharmacokinetic/pharmacodynamic (PK/PD) parameters for enrofloxacin (ENRO) calculated based on the recorded PD parameters and PK parameters derived from literature (ENRO 5 mg/kg bolus) or maximum enrofloxacin + ciprofloxacin (ENRO + CIPRO) plasma concentrations obtained in this study (ENRO 10 mg/kg via drinking water).

PK published parameters*		PD experimental parameters (μg/mL)		PK/PD		Reference values
C_max_	0.452 μg/mL	MIC_50_	0.03	C_max_/MIC_50_	15.0	>10
				C_max_/MPC_50_	1.8	n.d.
AUC_24_	4.36 μg/mL/h	MPC_50_	0.25	AUC_24_/MIC_50_	145.0	>125
				AUC_24_/MPC_50_	17.0	>14

ENRO + CIPRO maximum plasma concentration		PD experimental parameters (μg/mL)		PK/PD		Reference values
C_max_	0.148 μg/mL	MIC_50_	0.03	C_max_/MIC_50_	4.9	>10
		MPC_50_	0.25	C_max_/MPC_50_	0.6	n.d.

*Broome et al. (24); n.d = not determined.

## 4 Discussion

Antimicrobial resistance is a major challenge to livestock production and health safety. The main negative consequence of using ENRO in food producing animals such as rabbits, is the risk of selecting resistant bacteria. Moreover, beyond the risk of treatment failure, reduced ENRO susceptibility to *P. multocida* also represents a major risk for humans due to the potential transfer of resistance genes and to the zoonotic potential of *P. multocida* strains (34, 35). Furthermore, ENRO’s metabolic conversion to CIPRO, the active molecule authorized for use in humans, increases the risk of cross resistance (36).

Despite its widespread use over the past few decades, recent epidemiological studies have documented a trend of *P. multocida* resistance to ENRO in rabbits, which has not yet raised significant concern (4, 11, 13, 14). However, since resistance emergence is inevitable, and with few new antibiotics on the horizon, it is crucial to preserve the effectiveness of current drugs. European regulatory guidelines promote the prudent use of antibiotics in meat-producing animals and encourage alternative strategies (e.g., improved management practices, vaccination programs, selection of genetically resistant animals, use of probiotics, bacteriophages, antibody) to minimize the use of drugs in infection management.

Despite this, antimicrobial therapies remain the primary approach under field conditions. Thus, optimizing dosage regimens for systemic antimicrobials is key to counteract drug resistance.

Water medication is preferred for treating intensively reared rabbits. It’s more effective than medicated feed, as sick animals typically continue drinking even with reduced appetite. This method suits both therapeutic and metaphylactic treatments, is resource-efficient, and can be performed by unskilled staff.

Fluoroquinolones exhibit concentration-dependent killing with a moderate-to-prolonged persistent post-antibiotic effect. Thus, higher drug plasma concentration peaks are associated with better therapeutic outcomes. In this study, the plasma concentration trend throughout treatment showed a steady-state with minor fluctuations, reaching a maximum concentration that is less than half of that achieved following a single half-dose bolus (24). In rabbits, coprophagy may contribute to sustaining this kinetic pattern by recycling unmetabolized drug from feces, enhancing bioavailability (37).

ENRO clinical breakpoints for *P. multocida* in rabbits are unavailable. However, the CLSI defines the same sensitivity and resistance values against ENRO in chickens, turkeys (28), cattle, and pigs (29), which can partly justify using these breakpoints to interpret the results obtained in rabbits. Based on this assumption, all analyzed strains, from both healthy and untreated sick animals, were sensitive to ENRO and similar MIC and MBC values further confirm ENRO’s strong bactericidal activity against *P. multocida*. These findings, consistent with previous studies (4, 11, 13, 14), support ENRO’s continued effectiveness despite its long-term use in rabbits. However, visual inspection of the MIC distribution reveals a bimodal trend, with a lower frequency at VETCAST’s epidemiological cut-off value for ENRO (0.06 μg/mL) (38). Despite the small sample size, this may represent an early warning of potential emerging resistance (15).

To our knowledge, this is the first study examining ENRO’s antimicrobial potency against *P. multocida* from naturally infected food-production rabbits, thus precluding the possibility of direct comparisons within this rabbit category. Furthermore, comparative evaluations with data from other animal species are not relevant for the purpose of this study. However, similar results were found in pet rabbits with snuffles, where 62 *P. multocida* strains showed 100% susceptibility to ENRO, with MIC_50_ and MIC_90_ values of 0.013 and 0.079 μg/mL, respectively, and a bimodal MIC distribution (4).

Effective therapy is crucial for reducing symptoms, limiting bacterial spread, and controlling antibiotic resistance. ENRO is one of the most effective drugs for controlling the clinical signs of pasteurellosis in rabbits and it is approved for use in food-producing animals. Studies show that ENRO treatment (≥5 mg/kg, 10–20 days) via parenteral or drinking water routes can improve or cure clinical signs in naturally or experimentally infected rabbits (8–10, 39, 40). However, it may not eradicate pathogens persisting in areas such as the middle ear or turbinates (areas considered reservoirs), where effective ENRO concentrations might not be reached (39, 41).

Consequently, ENRO’s effectiveness in curing rabbit pasteurellosis remains uncertain, potentially offering only temporary symptom relief with the possibility of recurrence post-treatment.

In this study, despite the presence of ENRO-sensitive strains, the therapy appeared only partially effective, improving clinical conditions in less severely affected rabbits, but not inducing significant symptomatic changes in those with severe respiratory disorders, with two fatalities. The poor therapeutic response in rabbits with advanced disease stages, can be attributed to severe, irreversible lung damage caused by *P. multocida*, the shift of resident bacteria to less ENRO-sensitive opportunistic pathogen, and debilitation. In the latter case, the drug-induced bacterial reduction may be insufficient to support natural immune-mediated recovery.

A further explanation for the poor and variable clinical outcomes could be due to the drug administration via the drinking water, which affects the rabbits drug exposure (23). Indeed, despite uniform water consumption, individual plasma concentrations varied, suggesting that disease-related absorption differences rather than drinking behavior, might account for this variability.

ENRO pharmacokinetics can be altered in experimentally infected animals, such as those with *Escherichia coli* infections or endotoxiemia induced by *E. coli* endotoxin, as observed in studies on chickens and rabbits, respectively (42, 43). Additionally, feed presence alters fluoroquinolone absorption in many species (10, 44). Sick rabbits may reduce food intake and modify coprophagic habits, potentially altering the quantity and quality of gastric contents (37), which could modulate ENRO bioavailability.

Given these factors, the persistence of *P. multocida* in nasal swabs post treatment is not unexpected. Furthermore, no significant differences in ENRO susceptibility were found between pre-and post-treatment strains, suggesting that the approved treatment does not alter ENRO sensitivity within the *P. multocida* population of treated rabbits.

As expected, the recorded MPCs were significantly higher than the corresponding MICs (15), indicating the concentration below which any first-step ENRO-resistant clones may proliferate and replace the wild-type strain. It is important to mention that MPC values can vary by strain and organism, exhibiting more variability than MICs for a given bacterial strain-antibiotic combination (45). For instance, 73 *P. multocida* isolates from swine showed MPC values <0.25 μg ENRO/mL (46), while those from buffalo calves averaged 1.5 μg/mL (47).

In the present study, the average pre-treatment MPC value (0.26 μg/mL) indicates a still borderline sensitive bacterial population, with 3/10 strains showing intermediate sensitivity (0.5 μg/mL). In contrast, post-treatment MPC (0.5 μg/mL) reflects reduced sensitivity, with 3/9 strains exhibiting borderline resistance (1.0 μg/mL) (29). The higher post-treatment MPC_90_ and unchanged MPC_50_ further suggest that drug exposure may have led to the emergence of a mutant subpopulation in some animals, which may have prevailed over the wild-type strains.

While MPC alone does not resolve all resistance issues, it is a valuable tool for assessing the potential for resistance selection to fluoroquinolones, especially when combined with MSW to optimize dosing (15). The MSW spans from the wild population’s MIC to the first-step mutant’s MIC (i.e., MPC). Within this range, mutant pathogens gain a growth advantage over fully susceptible bacteria and may proliferate. Therefore, to prevent resistance, dosing regimens should be designed to minimize the time bacterial populations spend within the MSW during treatment (15).

The MSW size for resistance selection has not yet been defined; however, a lower MPI (MPC/MIC ratio) is generally associated with a reduced potential for resistant strain enrichment. For concentration-dependent antimicrobials, the MPC/MIC ratio is specific to the drug-pathogen pairing. For ENRO-*P. multocida*, MPC/MIC ratios range from 27 to 30 in buffalo calf isolates (47, 48), while MPC_50_/MIC_50_ and MPC_90_/MIC_90_ values of 4–8 were found in swine isolates (46).

In this study, the ENRO MPC/MIC ratio was relatively low for pre-treatment isolates (ratio = 9) but doubled post-treatment (ratio = 18). Individual post-treatment analysis of the MSW revealed even higher ratios (16.7–62.5), suggesting bacterial population modifications in some treated subjects.

Individual plasma analysis showed ENRO concentrations exceeding MIC in most animals (7/9, 78%), typically associated with positive therapeutic outcomes. However, 45% of subjects maintained concentrations within MSW limits throughout treatment, risking resistance selection. Only two subjects had plasma concentrations above the MSW upper threshold.

The transformation of ENRO into CIPRO can enhance therapeutic efficacy due to their additive activity (49). Therefore, the simultaneous presence of ENRO and CIPRO is expected to provide beneficial effects when ENRO is used to treat systemic *P. multocida* infections. However, in our study, even when considering ENRO + CIPRO plasma concentrations, the proportion of subjects maintaining concentrations within the MSW was unchanged. As a result, the potential for antimicrobial resistance development remained substantially unmodified.

Clinical and preclinical data suggest that the AUC_24_/MIC ratio is the key efficacy indicator for concentration-dependent antimicrobials, and it is recommended by VetCAST for dosage guidance and efficacy expectation (15). However, some authors also emphasize the importance of a high C_max_/MIC ratio in controlling resistance selection (50).

Without specific veterinary PK/PD indices, human medicine values are used, as they may have generic cross-species validity (15). For fluoroquinolones, an AUC_24_/MIC ratio > 125 and a C_max_/MIC ratio > 10 are used to predict therapy success against Gram-negative infections and control resistance in human and animal species (51–53). Additionally, an AUC_24_/MPC value >14 has been proposed for controlling enrichment of resistant mutants, based on the potentially more accurate prediction of resistance selection by MPC-based PK/PD indices (50).

Integrating PD data with PK parameters of ENRO administered via oral bolus at the dose of 5 mg/kg (24) meets proposed PK/PD criteria for both efficacy and resistance control. However, using the mean highest plasma concentration (estimated C_max_) obtained in this study from drinking water administration, the C_max_/MIC_50_ index falls below target values. This suggests that the ENRO authorized dosage is inadequate for treating rabbit pasteurellosis via drinking water.

Given the kinetic behavior of ENRO, concentrations that meet the MICs of pathogenic *P. multocida* strains could likely be achieved in the lungs (including alveolar macrophages, interstitial fluid, pulmonary epithelial fluid) (54–57) but not in other less accessible sites colonized by the bacteria. As a result, while improvements in respiratory symptoms may be observed, the overall exposure of the *P. multocida* population to ENRO may be insufficient for pathogen elimination, potentially facilitating the selection of resistant mutants in a high percentage of treated animals.

## 5 Conclusion

Although the results of this study are based on a limited number of rabbits and bacterial strains, they provide useful information on the use of ENRO for treating pasteurellosis under field conditions with approved drinking water medication, following standard husbandry practices.

Notably, the high individual variability in plasma drug concentration reveals a possible significant role that the disease may play in modifying the bioavailability of ENRO, thus affecting the exposure of *P. multocida* to the drug. Moreover, the MPC-based and MSW approaches suggest the possibility of acquired resistance, drug-induced changes in the *P. multocida* population, with a concerning 45% of subjects exhibiting a high potential for drug resistance selection.

Overall, these findings indicate that the currently authorized dosing regimen for treating rabbit pasteurellosis with ENRO via drinking water may be inadequate. It is therefore recommended to optimize the drug dosage regimen (dose and/or treatment schedule) to ensure that the effective dosage is well-tolerated while also controlling resistance selection, thus extending the clinical utility of ENRO.

However, due to the unique physiological characteristics of the rabbit’s gastrointestinal system the aggressive use of ENRO could be precluded in this species. To improve treatment effectiveness, limit the spread of resistance genes, and promote the responsible use of this antimicrobial in rabbits intended for meat production, ENRO therapy should always be complemented by additional infection control measures, such as improved herd management, better animal welfare practices, and the use of vaccines or alternative treatments.

## Data Availability

The raw data supporting the conclusions of this article will be made available by the authors, without undue reservation.
